# Mesenchymal Stem Cell-Derived Extracellular Vesicles for the Treatment of Bronchopulmonary Dysplasia

**DOI:** 10.3389/fped.2022.852034

**Published:** 2022-04-04

**Authors:** Yufeng Xi, Rong Ju, Yujia Wang

**Affiliations:** ^1^Department of Neonatology, Chengdu Women’s and Children’s Central Hospital, School of Medicine, University of Electronic Science and Technology of China, Chengdu, China; ^2^Department of Dermatology, State Key Laboratory of Biotherapy, West China Hospital, Sichuan University, Chengdu, China

**Keywords:** extracellular vesicles, therapy, chronic respiratory disease, mesenchymal stem cell, bronchopulmonary dysplasia (BPD)

## Abstract

Bronchopulmonary dysplasia (BPD) is the most common chronic respiratory disease in premature infants. However, there is a lack of effective treatment. Mesenchymal stromal cells derived extracellular vesicles (MSC-EVs), as nano- and micron-sized heterogeneous vesicles secreted by MSCs, are the main medium for information exchange between MSCs and injured tissue and organ, playing an important role in repairing tissue and organ injury. EVs include exosomes, microvesicles and so on. They are rich with various proteins, nucleic acids, and lipids. Now, EVs are considered as a new way of cell-to-cell communication. EVs mainly induce regeneration and therapeutic effects in different tissues and organs through the biomolecules they carry. The surface membrane protein or loaded protein and nucleic acid molecules carried by EVs, can activate the signal transduction of target cells and regulate the biological behavior of target cells after binding and cell internalization. MSC-EVs can promote the development of pulmonary vessels and alveoli and reduce pulmonary hypertension (PH) and inflammation and play an important role in the repair of lung injury in BPD. The regeneration potential of MSC-EVs is mainly due to the regulation of cell proliferation, survival, migration, differentiation, angiogenesis, immunoregulation, anti-inflammatory, mitochondrial activity and oxidative stress. As a new type of cell-free therapy, MSC-EVs have non-immunogenic, and are small in size and go deep into most tissues. What’s more, it has good biological stability and can be modified and loaded with drugs of interest. Obviously, MSC-EVs have a good application prospect in the treatment of lung injury and BPD. However, there are still many challenges to make MSC-EVs really enter clinical application.

## Introduction

Bronchopulmonary dysplasia (BPD) is the most common chronic respiratory disease in premature infants and low birth weight infants with high morbidity and mortality (1). With the development of perinatal medicine, the survival rate of low-birth-weight infants (LBWIs) and very low birth weight infants (VLBWIs) increased obviously, and the incidence of BPD increased year by year. Clinical epidemiologic studies show that the incidence of BPD in very premature infants is about 40% and increases with the decrease of gestational age (2). The mortality of BPD is high in the early stage, and the adverse outcomes in the respiratory system, circulatory system and even nervous system in the late stage, which seriously affect the survival rate and quality of life (3, 4). The clinical treatment of BPD has become a great challenge in perinatal and neonatal field. The pathogenesis of BPD is not clear at present, risk factors include preterm birth, fetal growth restriction, maternal smoking, mechanical ventilation, oxygen poisoning, infection, inflammation, patent ductus arteriosus (PDA), genetics, late surfactant deficiency, and impaired angiogenesis (5–13). Treatments for BPD include respiratory management, circulation management, nutritional support, and medication, including pulmonary surfactant, caffeine, glucocorticoid, diuretics, docosahexaenoic acid, and bronchodilator, however, the efficacy and safety need to be further explored (14–16). So far, there is no effective therapy to prevent or treat the development of lung injury, and therefore the research of new therapy is urgent.

In the last decade, pre-clinical studies and clinical studies indicate that therapies with mesenchymal stromal cells (MSCs) offer a new therapeutic approach for the prevention of BPD (17, 18). As the research continues, it is found that stem cells play their role mainly through extracellular vesicles (EVs) and other paracrine signal transduction (19, 20). Now, EVs are considered as a new way of cell-to-cell communication (21). They are present in biological fluids and are involved in many physiological and pathological processes. Here we review the progress in the treatment of BPD with mesenchymal stem cell extracelluar vesicles (MSC-EVs), with a view to bring new hope for the treatment of BPD.

## Advantages of Mesenchymal Stromal Cells Derived Extracellular Vesicles

Since MSCs were first reported as being derived from human bone marrow (BM) in 1999, they have been isolated from multiple tissues, including adipose tissue, amniotic fluid, umbilical cord blood, placental amnion, and placenta (22). MSCs are pluripotent and highly self-renewing stem cells derived from the mesoderm. They can promote the survival and repair of damaged cells, by being induced to differentiate into corresponding tissue cells and by regulating inflammation and immune response. What’s more, they can also promote the regeneration of damaged tissues by paracrine. Animal experiments showed that MSCs transplantation could prevent the growth stagnation of pulmonary vessels and alveoli, improve the simplification of alveolar structure and the abnormal development of pulmonary microvessels in BPD, and reduce pulmonary fibrosis (23, 24). MSCs are currently in clinical trials for the treatment of premature infants with BPD, and have achieved good curative effect (25, 26). Although MSCs have improved the physiological function of recipient lungs after treatment, some preclinical studies have pointed out that there are no large number of donor cells transplanted into the lungs, and the therapeutic effect of MSCs depend on its paracrine rather than cell replacement (27, 28). Hence, there is still a long way to go for mesenchymal stem cells to be used in clinical treatment.

**FIGURE 1:**
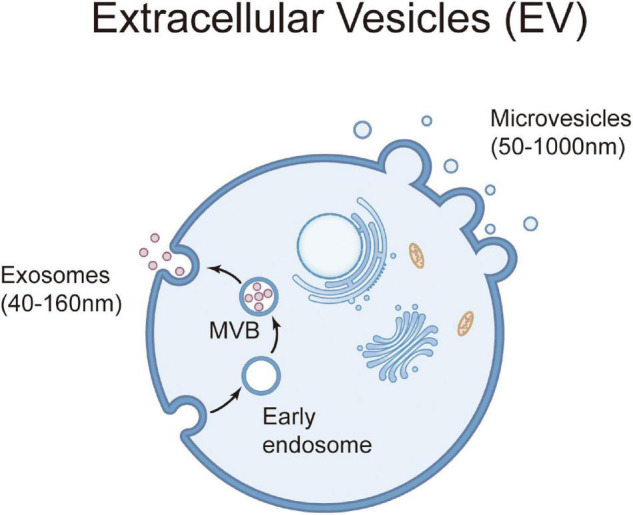
Generation and release of extracellular vesicles. Extracellular vesicles are a heterogeneous group of cell-derived membranous structures comprising microvesicles (50 nm–1 μm diameter) and exosomes (40–160 nm diameter). Microvesicles are shed from the plasma membrane, while exosomes originate from the endosomal system. Exosomes are secreted from cell to extracellular space by exocytosis after fusion of multivesicular body (MVB) (formed by endocytic vesicles) with plasma membrane.

Extracellular vesicles, a collective term covering various subtypes of cell-released, membranous structures, including exosomes, microvesicles, microparticles, ectosomes, oncosomes, apoptotic bodies, and so on, are delimited by a lipid bilayer and cannot replicate (29). Exosomes are of endosomal origin and in a size range of ∼40–160 nm in diameter, while microvesicles are vesicles generated by the direct outward budding of the plasma membrane in the size range of ∼50 nm–1 μm in diameter (30) (Figure 1). EVs are rich with various proteins, nucleic acids, and lipids. The main marker proteins of exosomes are CD9, CD63 and CD81, but there is no specific marker for microvesicles (30–32) (Table 1). MSC-EVs are nano- and micron-sized heterogeneous vesicles secreted by MSCs, which play an important role in repairing tissue and organ injury and is the main medium for information exchange between MSCs and injured tissue and organ (33). MSC-EVs, especially the smaller subcategory MSC-exosomes, may be superior to maternal cell therapy. MSC-exosomes have lower immunogenicity than its parent cells and can be modified to enhance bioavailability and cell targeting (34, 35). They can be used for cryopreservation without loss of activity and is convenient for drug preparation. Based on the above advantages, more and more scholars have proposed to use EVs as drug delivery carriers for targeted therapy (36). Therefore, MSC-EVs have become a hot spot in the treatment of BPD at home and abroad, but they are limited to animal experimental study at present.

**TABLE 1:** Characteristics of extracellular vesicles.

	Exosomes	Microvesicles
Biogenesis	Start with endocytosis, accumulate intraluminal vesicles of multivesicular bodies, and then release to extracellular spaces through exocytosis.	Released by budding and shedding from the plasma membrane of activated cells.
Size(nm)	40–160	50–1000
Contents	Nucleic acids (mRNA, microRNA, ssDNA, dsDNA, etc.), proteins, lipids, etc.	Nucleic acids (mRNA, microRNA, ssDNA, dsDNA, mitochondrial DNA, etc.), proteins, lipids, etc.
Marker	Tetraspanins (CD9, CD63, CD81, etc.)	No consensus marker

## The Role of Mesenchymal Stromal Cells Derived Extracellular Vesicles in the Treatment of Bronchopulmonary Dysplasia

**FIGURE 2:**
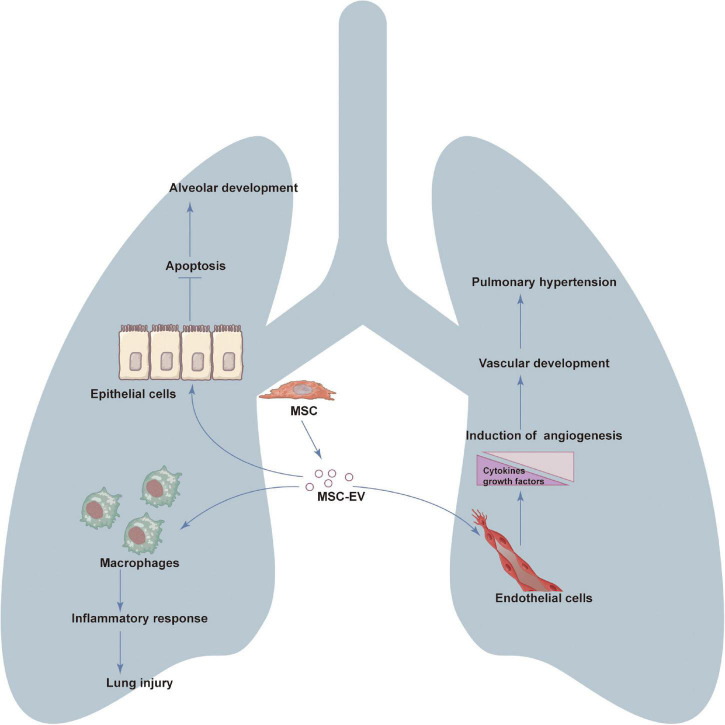
The role of mesenchymal stromal cells derived extracellular vesicles (MSC-EVs) in the treatment of bronchopulmonary dysplasia (BPD). MSC-EVs can promote the development of pulmonary vessels and alveoli and reduce pulmonary hypertension (PH) and inflammation and play an important role in the repair of lung injury in BPD. The regeneration potential of EVs is mainly due to the regulation of cell proliferation, survival, migration, differentiation, angiogenesis, immunoregulation and anti-inflammatory.

The clinical application of MSC-EVs includes both as drug delivery carrier and as an alternative to MSCs-based tissue and organ regeneration therapy. MSC-EVs can promote the development of pulmonary vessels and alveoli and reduce pulmonary hypertension and play an important role in the repair of lung injury in BPD (Figure 2).

### Mesenchymal Stromal Cells Derived Extracellular Vesicles Promote the Development of Pulmonary Microvasculature

As described above, pulmonary microvascular dysplasia is one of the main pathological manifestations of BPD. Therefore, promoting pulmonary angiogenesis is the key to the improvement of pulmonary vascular dysplasia. Porzionato et al. (37) conducted a study to compare the protective effects of intratracheally (IT) administered MSCs vs. MSC-EVs in a hyperoxia-induced rat model of BPD. They concluded that both EVs and MSCs reduce hyperoxia-induced damage, with EVs obtaining better results in terms of alveolarization and lung vascularization parameters. In addition, a study by Rudolf K Braun et al. (38) found that daily intraperitoneal injection of MSC-derived exosomes protected alveolarization and angiogenesis in a newborn rat model of BPD induced by 14 days of neonatal hyperoxia exposure. *In vitro*, exosomes significantly increased tube-like network formation by human umbilical vein endothelial cell (HUVEC), in part through a vascular endothelial growth factor(VEGF)mediated mechanism.

In summary, daily intraperitoneal injection of exosomes increased blood vessel number and size in the lung through pro-angiogenic mechanisms. The results suggest that MSC-EVs can promote pulmonary microvasculature in experimental animals with BPD.

### Mesenchymal Stromal Cells Derived Extracellular Vesicles Promote the Development of Alveoli

The main pathological features of bronchopulmonary dysplasia in premature infants were hypoplasia of alveoli, decreased number, increased volume and simplified structure of alveoli. Over-death of alveolar type II epithelial cells is the key cause of alveolar hypoplasia. Many studies have shown that MSC-exosomes play an anti-apoptotic role in diseases closely related to apoptosis by regulating the apoptotic process. Yunfei Wu et al. (39) established hyperoxia-induced lung injury (HILI) rat models and RLE-6TN cell models, then treated them with BMSCs-exosomes. BMSCs-exosomes attenuated HILI and H2O2 induced RLE-6TN cell injury as evidence by alleviated lung cell injury, decreased TUNEL-positive cells, induced cell viability and declined apoptosis. In addition, Sushma Chaubey et al. (40) reported that early gestational MSC-exosomes treatment reverses alveolar injury, septal thickness and other morphometric alterations associated with hyperoxia-induced lung injury in the BPD mouse model. Ai et al. (41) found that MSC-EVs ameliorated hyperoxia-induced lung injury in a dose-dependent manner, and high-dose MSC-EVs ameliorated alveolar simplification and fibrosis. What’s more, MSC-EVs showed its beneficial effects on vascular growth and pulmonary hypertension. A meta-analysis (42) included eight articles on the treatment of BPD with MSC-EVs, concluded that alveolarization was improved by MSC-EVs (SMD −1.45, CI −2.08 to −0.82) with small EVs more consistently beneficial then small/large EVs. In conclusion, EVs from different MSC sources can effectively promote alveolar development and reduce lung injury.

### Mesenchymal Stromal Cells Derived Extracellular Vesicles Reduce Pulmonary Hypertension

Combined pulmonary hypertension (PH) is one of the important characteristics of BPD, the incidence is 19.4 ∼ 40.0%, which is closely related to the degree of BPD. The mortality of BPD with PH was significantly increased, which was an important cause of late death in BPD patients. Jingyi You et al. (43) found that human umbilical cord MSC- EVs were successfully absorbed by lung tissue after intratracheal administration, and remained in the lungs for at least 72 h. The results showed that human umbilical cord MSC-EVs not only could improve alveolarization and angiogenesis, but also could ameliorate pulmonary hypertension in a rat model of BPD meantime. Willis et al. (44) found that delivery of MSC-EVs improves core features of experimental BPD, restoring lung architecture, decreasing pulmonary fibrosis and vascular muscularization, ameliorating PH and improving exercise capacity. What’s more important, delivery of MSC-EVs may not only be effective in the immediate neonatal period to prevent the development of BPD but may provide beneficial effects for the management and potentially the reversal of cardiorespiratory complications in infants and children with established BPD. In view of the multiple roles of MSC-EVs in the treatment of BPD, MSC-EVs has broad application prospects in future.

Mesenchymal stromal cells derived extracellular vesicles treatment for animal BPD model and their relative mechanisms were summarized in the Table 2.

**TABLE 2:** Mesenchymal stromal cells derived extracellular vesicles (MSC-EVs) treatment for hyperoxia-induced rat model of bronchopulmonary dysplasia (BPD).

MSC-EVs	Dose	Effect	Mechanism	References
Umbilical cord MSC-EVs	0.64 × 10^10^	Reduced hyperoxia-induced damage	Promoting alveolarization and pulmonary vascular remodeling	(37)
Rat bone marrow MSC- exosomes	4.76 × 10^7^	Prevented disruption of alveolar growth, increased small blood vessel number, and inhibited right heart hypertrophy	Both anti-inflammatory and pro-angiogenic mechanism	(38)
Rat bone marrow MSC- exosomes	800 μg	Relieved lung injury	MiR-425 in exosomes suppressed HILI by targeting PTEN and upregulating the PI3K/AKT axis	(39)
Umbilical cord blood MSC-exosomes	2.4 μg	Resulted in robust improvement in lung, cardiac and brain pathology	TSG-6 in exosomes decreased proinflammatory cytokines IL-6, TNF-α and IL-1β and cell death	(40)
MSC-EVs	Unknown	Ameliorated hyperoxia-induced lung injury in a dose-dependent manner, and high-dose MSC-EVs ameliorated alveolar simplification, fibrosis.	MSC-EVs suppressed the transdifferentiation of AT2 cells by downregulating WNT5a.	(41)
Umbilical cord MSC-small EVs	80 μg	Restored alveolar structure and lung function, and ameliorated pulmonary hypertension	Improved alveolarization and angiogenesis by inhibiting PTEN and activating Akt signaling pathway	(43)
Umbilical cord MSC-small EVs	Corresponded to 1 × 10^6^ cell equivalents	Improved core features of experimental BPD, restoring lung architecture, decreasing pulmonary fibrosis and vascular muscularization, ameliorating PH and improving exercise capacity	Promoting alveolarization and angiogenesis	(44)
Umbilical cord blood MSC-EVs	20 μg	Ameliorated neonatal hyperoxic lung injuries, such as impaired alveolarization and angiogenesis, increased cell death, and inflammatory responses	EV mediated VEGF transfer	(46)
Umbilical cord MSC-EVs	10 μg	Inhibited lung inflammation, vascular remodeling and right heart failure, and reverses PH	Suppressed STAT3 activation in lung vascular cells and upregulated miR-204 levels	(49)
Umbilical cord MSC-exosomes and bone marrow MSC- exosomes	Corresponded to 1 × 10^6^ cell equivalents	Resulted in alleviation of inflammation, improvement of lung function and alveolarization, decrease in fibrosis and pulmonary vascular remodeling, and amelioration of pulmonary hypertension	Modulated the macrophage phenotype fulcrum, suppressing the proinflammatory “M1” state and augmenting an anti-inflammatory “M2-like” state.	(52)
MSC-EVs	Unknown	Ameliorating the impaired alveolarization and pulmonary artery remodeling	Promoted M2 macrophage polarization, and inhibited inflammatory response	(53)
Umbilical cord MSC-exosomes	Corresponded to 1 × 10^6^ cell equivalents	Restored alveolar architecture, blunted fibrosis and pulmonary vascular remodeling, and improved exercise capacity	Promoted M2 macrophage polarization though epigenetic and phenotypic reprogramming of myeloid cells.	(54)
Umbilical cord MSC-exosomes	Corresponded to 0.5 × 10^6^ cell equivalents	A promising restorative therapeutic approach for oxygen-induced thymic injury, thus promoting normal development of both central tolerance and adaptive immunity	Promoted T cell development to realize immune regulation	(55)

## The Mechanism of Mesenchymal Stromal Cells Derived Extracellular Vesicles in the Treatment of Bronchopulmonary Dysplasia

Many studies have shown that EVs mainly induce regeneration and therapeutic effects in different tissues and organs through the biomolecules they carry. As a natural macromolecular carrier, the surface membrane protein or loaded protein and nucleic acid molecules carried by natural EVs, such as mRNA and miRNA, can activate the signal transduction of target cells and regulate the biological behavior of target cells after binding and cell internalization.

### Promoting Angiogenesis

Mesenchymal stromal cells can secrete many kinds of growth factors associated with angiogenesis such as VEGF and store them in MSC-EVs. High-resolution isoelectric focusing coupled liquid chromatography tandem mass spectrometry, a non-targeted high-throughput proteomic method, was used to map the protein profiles of MSCs and MSC-exosomes in a recent study (45). The study found that MSC-exosomes contain many proteins related to angiogenesis, such as VE-cadherin, EGFR, FGF, PDGF. VEGF is the key regulator in regulating the growth, development, and repair of pulmonary vessels in the whole embryonic stage, fetal stage, and postnatal stage. It is also involved in the occurrence of neonatal hyperoxia lung injury. VEGF mediated angiogenesis is considered to be one of the mechanisms of MSC-exosomes in the treatment of BPD, which participates in the repair of endothelial cell injury by reducing endothelial cell apoptosis, promoting endothelial cell proliferation, reducing vascular endothelial cell permeability, and promoting angiogenesis. The therapeutic effect of MSC-exosomes on neonatal rat hyperoxia lung injury (HLI) model shows that the mechanism of MSC-exosomes in reducing neonatal rat HLI is mainly mediated by VEGF (38). A study compared the therapeutic efficacy of MSCs, MSC-EVs with or without VEGF knockdown, and fibroblast-derived EVs *in vitro* with a rat lung epithelial cell line challenged with H2O2 and *in vivo* with newborn Sprague-Dawley rats exposed to hyperoxia (90%) for 14 days. The study indicated that MSCs and MSC-EVs, but not the EVs derived from VEGF-knockdown MSCs or fibroblasts, attenuated hyperoxic lung injuries, such as impaired alveolarization and angiogenesis, increased cell death, and activated macrophages and proinflammatory cytokines. All in all, MSC-derived EVs are as effective as parental MSCs for attenuating neonatal hyperoxic lung injuries, and this protection was mediated primarily by the transfer of VEGF (46).

### Anti-apoptosis

Alveolar epithelial type II cell (AEC-II) are progenitor cells of lung epithelium, which can proliferate, differentiate, and repair lung injury. Besides, AEC-II can synthesize and secrete pulmonary surfactant and participate in innate immunity and immunomodulation. AEC-II play an important role in maintaining the structure and function of pulmonary alveoli and the homeostasis of local environment (47). AEC-II are the key target cells for BPD lung epithelial injury. Previous studies have shown that excessive apoptosis of AEC-II cells is the key cause of BPD pulmonary alveolar dysplasia (48). Recent studies have shown that MSC-EVs contains a variety of anti-apoptotic proteins, can play an anti-apoptotic role in diseases closely related to apoptosis by regulating the apoptosis process. *In vitro*, under hyperoxic conditions, the tube-like structure formation was improved in HUVECs, and the proliferation was increased, and the apoptosis was attenuated in MLE-12 cells treated with human umbilical cord MSC-EVs (43). There is little research on the anti-apoptotic effect of MSC-EVs in lung injury, and more experimental studies are needed to elucidate this mechanism.

### RNA Transfer

Mesenchymal stromal cells derived extracellular vesicles contain a variety of non-coding RNAs, such as microRNAs, long non-coding RNAs(lncRNAs). Many studies have shown that EVs play a biological role in the transmission of RNA in intercellular communication. The selective loading of specific RNA into exosomes protects it from the effects of extracellular nucleases, thus prolonging its half-life and enhancing its biological activity, exocrine body as its carrier can realize the exchange of genetic information and cell-to-cell communication. Lee et al. found that MSC-EVs are composed of miR-16, miR-21 and Let-7b pre-miRNA (49). In another study, bone mesenchymal stem cells (BMSC)-exosomes promoted miR-425 expression and attenuated hyperoxia-induced lung injury (HILI) and H2O2 induced RLE-6TN cell injury as evidence by alleviated lung cell injury, induced cell viability and declined apoptosis (39). Besides, when miR-425 was knocked-down, the protective role of BMSC-exosomes in HILI was also reduced. It is suggested that MSC-EVs may partially alleviate lung injury by RNA transfer in experimental animals with BPD.

### Immune Regulation and Inflammatory Response

One of the pathogenesis of BPD is the disorder of inflammatory reaction. MSC-EVs can regulate the out-of-control inflammatory response through immunoregulation (50, 51). Alveolar macrophages are critical mediator in the pulmonary immune response to BPD, which is involved in the initiation and the resolution of inflammation. Pulmonary macrophages have pro-inflammatory M1 like and anti-inflammatory M2 like effects and are important target cells of MSC-EVs. MSC-exosomes treatment induced pleiotropic effects on gene expression associated with inflammation and immune responses in hyperoxia exposed newborn mice (52). MSC-exosomes modulate the macrophage phenotype fulcrum, suppressing the proinflammatory “M1” state and augmenting an anti-inflammatory “M2-like” state, both *in vitro* and *in vivo*. Tumor necrosis factor alpha-stimulated gene-6(TSG-6), involved in MSC-exosomes immunoregulation, regulates inflammatory mediators such as Tumor necrosis factors-α(TNF-α) and interleukin-1β(IL-1β), and is one of the key factors of immunosuppression. TSG-6 has been shown to protect rats from lipopolysaccharide(LPS)-induced pulmonary inflammation and injury by inducing the phenotype of macrophages from pro-inflammatory M1 to anti-inflammatory M2 (40). In addition, TSG-6 siRNA-transfected MSC-exosomes abrogated the therapeutic effects of exosomes, suggesting TSG-6 as an important therapeutic molecule. Porzionato et al. (53). found that hyperoxia exposure reduced CD163-positive macrophages both in interstitial/alveolar and perivascular populations and MSC-EV prevented these hyperoxia-induced reductions. Further study suggested that intratracheally-administered EVs could be an effective approach to prevent/treat BPD, ameliorating the impaired alveolarization and pulmonary artery remodeling also in a long-term model. Willis et al. (54) found that MSC-EVs restored the apportion of alveolar macrophages in the hyperoxia induced lung injury and concomitantly suppressed inflammatory cytokine production. *In vitro and in vivo* studies revealed that MSC-EVs promoted an immunosuppressive in bone marrow-derived myeloid cells phenotype. A study (55) demonstrated that MSC-EVs treatment represents a promising restorative therapeutic approach for oxygen-induced thymic injury, thus promoting normal development of both central tolerance and adaptive immunity.

Hence, inflammatory response and immunoregulation may be an important mechanism for MSC-EVs to reduce lung injury in BPD experimental animals.

### Regulating Abnormal Mitochondrial Activity and Inhibit Oxidative Stress

Oxidative stress is one of the most predominant causes of BPD (56). Oxidative stress induced by hyperoxia exposure is regulated by the antioxidation *in vivo*. Due to the poor tolerance to hyperoxia, premature infants are also vulnerable to reactive oxygen species (ROS) mediated injury, which is more likely to cause lung injury and development retardation, so that oxidative stress participates in all links of the pathogenesis of BPD in premature infants. Mitochondria have been considered as a main ROS source and as a key intracellular buffer that protects against oxidant stress (57). Hyperoxia is known to inhibit pulmonary mitochondrial bioenergetic function and impair alveolar development in animal models of lung injury (58, 59).

Recent studies (52, 60) have reported that MSCs can release extracellular vesicles containing mitochondria, and they mediated mitochondrial transfer to macrophages. The protection of MSC-EVs against lung injury can be attributed to the interaction between mitochondria and alveolar macrophages. Mitochondrial transfer increases the bioenergy and function of macrophages. MSC-EVs may partially reduce lung injury in BPD experimental animals through mitochondrial transfer. Phinney et al. (60) reported that bone mesenchymal stem cells (BMSCs) manage intracellular oxidative stress by targeting depolarized mitochondria to the plasma membrane *via* arrestin domain-containing protein 1-mediated microvesicles. In addition, the vesicles are then engulfed and re-utilized *via* a process involving fusion by macrophages, resulting in enhanced bioenergetics. Furthermore, this study also showed that MSCs simultaneously shed microRNA-containing exosomes that inhibit macrophage activation by suppressing Toll-like receptor signaling, thereby de-sensitizing macrophages to the ingested mitochondria.

In summary, MSC-EVs can transfer mitochondria, microRNAs, and protein to macrophages to improve mitochondrial dysfunction and oxidative stress damage.

## Prospect and Challenge

As mentioned above, more and more experimental studies have found MSC-EVs can promote the development of pulmonary vessels and alveoli and reduce pulmonary hypertension and inflammation and play an important role in the repair of lung injury in BPD. MSC-EVs are more superior than MSCs is mainly manifested in: First, MSC-EVs are derived from cells and have non-immunogenic, which can avoid tumor transformation and immune response activation, so more safe; Second, as a new type of cell-free therapy, MSC-EVs are small in size and go deep into most tissues; Third, it has good biological stability and can be modified and loaded with drugs of interest; Fourth, MSC-EVs maintain good biocompatibility and can carry many types of biomolecules; Fifth, the surface specific receptors or antibodies can be processed to deliver therapeutic molecules to target cells. Obviously, MSC-EVs have a good application prospect in the treatment of lung injury and BPD. Although many scholars have conducted in-depth research on the components and functions of MSC-EVs, the mechanism of them in disease has not been completely cleared and needs to be further explored. Many studies have shown that EVs mainly induce regeneration and therapeutic effects through the biomolecules they carry, such as protein, nucleic acid molecules, which can regulate the biological behavior of target cells, for example cell proliferation, survival, migration, differentiation, angiogenesis, immunoregulation and anti-inflammatory, mitochondrial activity, oxidative stress.

At present, the clinical research of mesenchymal stem cells in the treatment of bronchopulmonary dysplasia are widely carried out all over the world, but the clinical research on extracellular vesicles of mesenchymal stem cells is very limited. However, there are still many challenges to make MSC-EVs really enter clinical application (61–63). At first, the research evidence of MSC-EVs in the treatment of BPD almost comes from preclinical studies. Next, the mechanism of MSC-EVs in the treatment of BPD is still unclear. What’s more, the preparation process of MSC-EVs lacks unified standards (64), such as MSC-EVs source, purification methods of MSC-EVs, composition of MSC-EVs, MSC-EVs purity. For the quality control of extracellular vesicles, we can refer to The International Society for Extracellular Vesicles (ISEV) proposed Minimal Information for Studies of Extracellular Vesicles (“MISEV”) guidelines for the field in 2014 and 2018 (29, 65). Most important of all, the appropriate children, treatment time, appropriate pathway, and effective dose of MSC-EVs need to be determined (66–68). The main delivery methods of extracellular vesicles are intratracheal administration, nasal inhalation, Intraperitoneal injection, and intravenous administration (37, 38, 69, 70). As for which pathway of administration is better and how much are the dosage, further research is still needed. The influence of the origin of EVs on their distribution in organisms also needs to be discussed. The holistic analysis (71) has shown that, regardless of the source or size of EVs or the species into which the EVs were delivered, EVs typically accumulate in a restricted number of organs: liver, lungs, kidneys, and spleen. The physiological or pathological state of the donor affects the functional plasticity of the EVs. The microenvironment of cells and the biological processes occurring in cells will affect the contents contained in exosomes. For example, exosomes secreted by mesenchymal stem cells under hypoxia contain high levels of HIF-1a and microRNAs that promote angiogenesis, such as mir-210, mir-216 (72, 73). In addition, hypoxia also promote the release of exosomes by MSCs. EVs have high heterogeneity, including source heterogeneity, size heterogeneity, content heterogeneity and functional heterogeneity. These heterogeneities also affect the function of extracellular vesicles (30). In the future, multidisciplinary comprehensive cooperation and large sample research are needed to accumulate more data and experience, and repeatedly evaluate the safety and effectiveness of treatment, so as to provide sufficient theoretical basis for clinical trials.

## Conclusion

In conclusion, many related studies have shown the great prospect of MSC-EVs in the treatment of lung injury, which is expected to become an effective treatment for BPD. However, there are still many difficulties in its real application in clinic, and more research and exploration are needed.

## Author Contributions

YX: conception and design. YW and RJ: administrative support. All authors contributed to the manuscript writing and final approval of the manuscript.

## Conflict of Interest

The authors declare that the research was conducted in the absence of any commercial or financial relationships that could be construed as a potential conflict of interest.

## Publisher’s Note

All claims expressed in this article are solely those of the authors and do not necessarily represent those of their affiliated organizations, or those of the publisher, the editors and the reviewers. Any product that may be evaluated in this article, or claim that may be made by its manufacturer, is not guaranteed or endorsed by the publisher.
